# Diversity of leaf anatomy within a single leaflet and between leaflets of four *Butia* (Arecaceae, Arecoideae) species

**DOI:** 10.3897/phytokeys.180.66018

**Published:** 2021-08-03

**Authors:** Larry R. Noblick, Bruno F. Sant’anna-Santos

**Affiliations:** 1 Montgomery Botanical Center, 11901 Old Cutler Road, 33156, Miami, Florida, USA Montgomery Botanical Center Miami United States of America; 2 Departamento de Botânica, Universidade Federal de Paraná, Curitiba, Paraná, Brazil Universidade Federal de Paraná Paraná Brazil

**Keywords:** anatomy, environmental variation, interspecific variation, key

## Abstract

This paper is an investigation into how the anatomy changes within a leaflet and between the leaflets of a single leaf blade of *Butia*. Four species of *Butia* were studied: *Butiaparaguayensis*, *B.eriospatha*, *B.yatay* and *B.odorata*. Changes in the anatomical characters are important because some have been used in keys to help separate the species. Recently, anatomical mid-rib characters were used in a key to separate species of *Butia*. We found that characters, such as abaxially projected or rounded mid-rib fibrous ring or number and arrangement of accessory bundles, do change within a single leaflet or between the leaflets of a single leaf blade. Growing conditions and leaf developmental maturity are also important factors that influence leaflet anatomy and may cause one to be misled in an identification key based on anatomical characters. We re-emphasize the importance of always sampling from the same part of the leaf, to have a broader sampling, be attentive to the environmental condition and health of the plant from which you are sampling and to consider population differences.

## Introduction

Palms (Arecaceae) are an easy family of plants to identify to family and are divided into five subfamilies: Calamoideae, Nypoideae, Coryphoideae, Ceroxyloideae and Arecoideae (Dransfield et al. 2008). Palm leaves come in many different shapes: fan, entire or pinnate. Fan-like leaves, such as palmate or costapalmate leaves, are identified with the subfamilies Coryphoideae and the Calamoideae, in particular, the Lepidocaryeae tribe. All of the other subfamilies including most of the other Calamoideae have entire or pinnate leaves. One of the genetically most diverse subfamilies is the Arecoideae with 10 tribes, amongst which belong the tribe Cocoseae. Cocoseae are further divided into three subtribes: Attaleinae, Bactridinae and Elaeidinae. The non-spiny Attaleinae is the subtribe to which belongs the well-known coconut (*Cocos*) and also *Butia*, the genus we will be focusing on in this paper.

Leaflet anatomy has been advocated as an alternative method for the identification of palm species. The methodology is simple – choose a middle leaflet and section its centre (or center in American English). Tomlinson (1961) hinted that leaf anatomy might be useful in palm identification. It was used to identify species of *Syagrus* (Glassman 1972; Noblick 2013, 2017; Firmo et al. 2021) and, more recently, species of *Butia* (Sant’Anna-Santos et al. 2015, 2018). Sanin and Galeano (2011) used leaf anatomy to distinguish species of *Ceroxylon* Bonpl. ex DC. and Noraini et al. (2012) to distinguish species of *Johannesteijsmannia* H.E. Moore. Pinedo et al. (2016) wrote a key to the species of *Allagoptera* and Vianna et al. (2017) wrote a key to the species of *Acrocomia* using leaflet anatomy. Finally Guevara et al. (2011) was able to distinguish the three genera of the subtribe Mauritiinae, but unfortunately, was unable to separate the species using leaflet anatomy.

While Glassman (1972) and Noblick (2013, 2017) found plenty of characters in the leaflet margin to distinguish species of *Syagrus*, finding differences in the leaflet margins to distinguish *Butia* species has been challenging, even while *Butia*’s isolateral, nearly mirrored anatomy is one of its most compelling and distinguishing generic characters. Although differences in *Butia* leaflet marginal anatomy have failed to distinguish most species, Sant’Anna-Santos et al. (2015, 2018) discovered that there were important differences found in the mid-ribs, an issue previously stated by Glassman (1972). They used these characters to confirm the dissimilarities between *Butiacapitata* (Mart.) Becc. and *Butiaodorata* (B.Rodr.) Noblick (Sant’Anna-Santos et al. 2015) and also to construct a key to the various other *Butia* species (Glassman 1972; Sant’Anna-Santos et al. 2018). Recently, the leaflet anatomy was employed in the description of a new species of *Butia*, *Butiabuenopolensis* (Sant’Anna-Santos 2021).

After examining leaflet cross-sections of over 100 accessions of *Butia* from the living collections at Montgomery Botanical Center, the first author began to develop some concerns. What if the exact middle leaflet is not selected for study? How quickly does palm leaflet anatomy change depending on which “middle” leaflet is chosen (closer to the base or closer to the leaf apex)? Does the leaflet anatomy change much depending on where the section is made along the leaflet? Does the anatomy change from one side of the leaf blade to the other? Does the leaflet anatomy change from between a more mature and less mature or lesser developed specimen of the same species? Are there differences in the anatomy of the same species from one population to another? These are questions that have not been adequately addressed by those using leaflet anatomy for the purpose of identification and that includes ourselves.

## Materials and methods

### Plants examined

**Figure 1.**
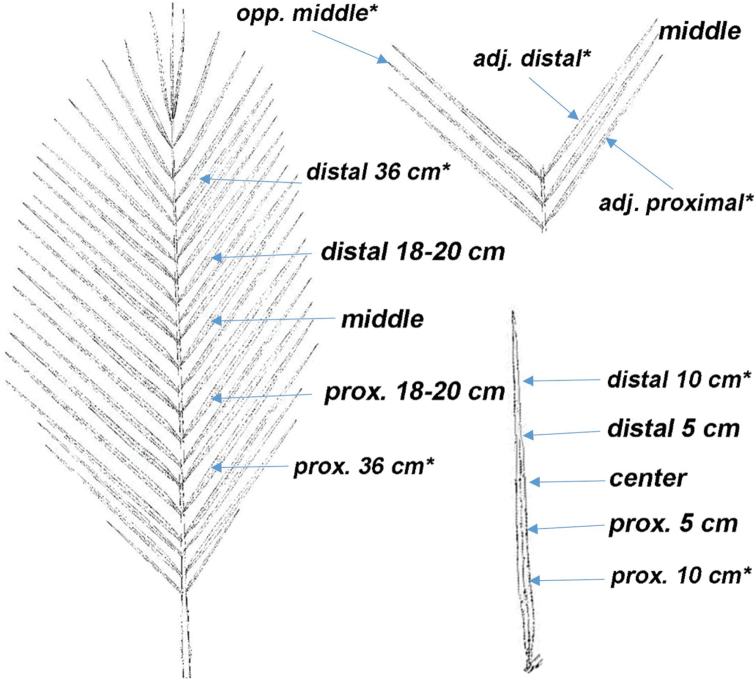
Leaf diagram showing which leaflets of the leaf and which sections of each leaflet that were sampled. * = regions that were only sampled in *Butiaparaguayensis*.

Fresh material was used to prepare 67 slides from four leaves; one leaf from each of the four *Butia* species. The specimens, sampled in this study, came from the living collections at Montgomery Botanical Center (MBC), Miami, FL. *Butiaparaguayensis* (Barb.Rodr.) L.H.Bailey (MBC accession 20020856*C) was grown from seed collected from San Estanislão, Paraguay and was sampled more thoroughly than the others. Figure 1 shows the regions from which our samples were taken for this study and an asterisk indicates those leaf regions that were only sampled in *Butiaparaguayensis*. In *B.paraguayensis*, each of eight leaflets was sectioned in five different places: their centre, 5 cm to either side and 10 cm to either side for a total of 40 slides.

After much sectioning, it was reasoned that, if one simply “eye-balls” the middle of a leaf, one is not likely to select a middle leaflet more than 20 cm to either side of the true middle of a leaf, nor section that leaflet by more than 5 cm to either side of its centre. Therefore, only three leaflets were sampled from each of other three *Butia* species, which included *B.yatay* (Mart.) Becc., *B.eriospatha* (Mart. ex Drude) Becc. and *B.odorata* (Barb.Rodr.) Noblick. These three species were chosen, because they all have aboveground stems like *B.paraguayensis* and live, wild collected specimens were readily available at MBC. Most of these species are also widely distributed geographically, allowing us to test for some degree of variation in morphological and anatomical characters. *Butiaeriospatha* (92271*E) was grown from seed collected from Paraná, Brazil. *Butiaodorata* (20060233*E) was grown from seed collected from Rocha, Uruguay and *Butiayatay* (20040309*C) was grown from seed collected from Batel, Argentina. The leaflets were collected from the middle and 20 cm to either side of the middle. Each of these leaflets was sampled in three places: the centre and 5 cm to either side of it for a total of nine slides for each species and a total of 27 slides.

### Anatomical preparation

Several methods for hand sectioning are covered in Noblick (2013, 2017). For this study, the following equipment was used: a hand microtome, a sharp knife, a straight razor, a double-sided razor blade, a small artist’s brush, a squirt water bottle, a watch glass, a sharpening stone and a carrot. The hand microtome was purchased from homesciencetools.com, Billings, MT, U.S.A. We used a Dovo Straight Razor 3” Full Hollow Ground Carbon Steel Blade for sectioning. Finally, to keep a razor-sharp edge, the Dia-sharp 3 micron 8000 mesh (DMT D8EE 8” Extra Extra Fine Diamond Stone) (DMTsharp.com or Diamond Machining Technology, Marlbourough, MA, U.S.A) was found to be an excellent choice. Using it frequently between sectioning maintains the razor sharpness required for making clean, thin sections.

### Preparing the section

**Figure 2.**
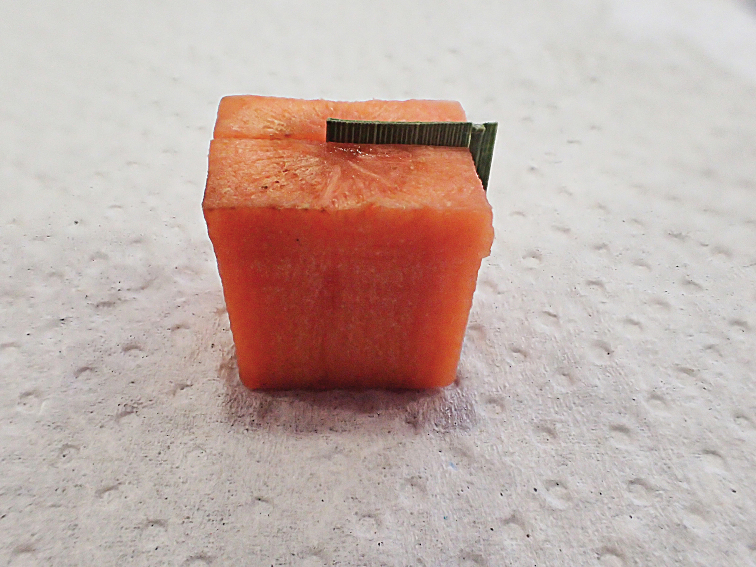
Carrot cube with leaflet sample secured within the carrot slit. Mid-rib of leaflet sample orientated parallel and adjacent to the side of the cube, ready to be placed in the hand-held microtome for sectioning.

A piece of carrot is cut into a small cube that will fit in the hand microtome as described in Noblick (2017). A deep perpendicular vertical slit is cut in the top of the carrot cube with the double-sided razor blade. The slit in the carrot cube is used to secure the leaflet vertically. If the leaf is stiff and coriaceous, you can mount the specimen with a few mm exposed above the top of the carrot cube with the mid-rib mounted adjacent and parallel to the cube’s side (Figure 2). The stiffer, thicker *Butia* leaflets mounted thus allow us to make thinner sections quicker with the straight razor without having to sort through carrot debris. If the specimen is thin and membranaceous, then the specimen must be mounted within the carrot for better support. The carrot cube is then secured in the hand microtome and adjusted down until it is just below the microtome plate. Lubricate the specimen with a drop of water and slide the straight razor across the microtome in a slicing movement, while pressing the side of the blade firmly against the plate. Adjust the microtome up by about a quarter of a turn after each section. Periodically, re-sharpen the blade using the Diamond Stone and water. Keep the specimen lubricated with water. After obtaining one to three sections, tease the sections into the water-filled watch glass using the small artist’s brush. Any more than that and you risk cutting the sections you just obtained. Keep sectioning until you obtain four to six good specimens to place on your slide.

### Preparing the slide

Glass slides with one side frosted for labelling, glass cover slips, artist’s brush, a dropper bottle of 1:1 glycerine and water solution and a dissecting needle are needed for slide preparation. Confirm that the glass slide and glass cover slip are clean before using. Label the frosted portion of the slide and spread a drop or two of the 1:1 glycerine and water solution on to the slide. While looking through the dissecting microscope, select the best sections from the watch glass with the narrow artist’s brush and transfer them into the 1:1 glycerine droplet on the slide. After placing a number of the sections on the slide (ca. 4–6), cover the sections with a glass cover slip. Place one edge of the cover slip at the edge of the glycerine droplet on the slide and gently lower it in place over the sections by placing the dissecting needle tip on its side under the other edge of the cover slip. While slowly pulling out the needle as the cover slip lowers into place, most of the air bubbles should exit from under the cover slip on the side of the exiting needle.

For the collection from Tapes, the samples followed the additional protocol proposed by Sant’Anna-Santos et al. (2015) including the softening and double staining with safranin and astra blue (Kraus and Arduin 1997).

### Photography

The glass slide is now placed under a 40×–2000× Trinocular Biological Compound Microscope available from Amscope (model T490B) and photographed under the 10× objective (100× magnification). Images were taken with a 5 Mb AmScope digital camera. The images were cleaned of background spots, adjusted for brightness and sharpened, if necessary, using Adobe Photoshop. If the entire mid-rib or leaf margin did not fit into the field of view, adjacent images were photo-merged using the automatic photo-merge capabilities of Adobe Photoshop. A stage micrometer was used to apply a scale to each image.

### Characters defined

**Figure 3.**
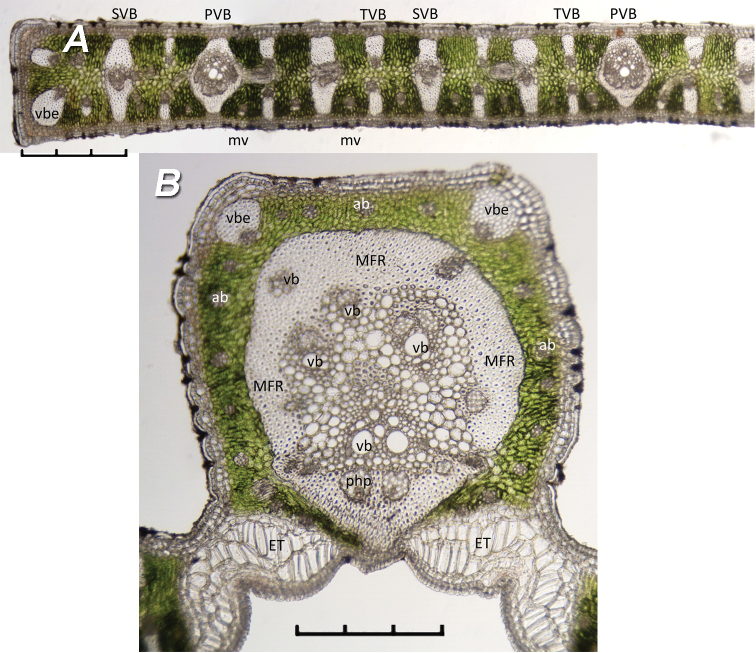
Anatomical characters of leaflet cross-sections **A***Butiaodorata* leaflet margin showing isolateral “mirrored” anatomy **B***B.yatay* midrib. **ab** = accessory bundle; **ET** = expansion tissue; **MFR** = mid-rib fibrous ring; **mv** = miniveins; **php** = phloem pole; **PVB** = primary vascular bundle; **SVB** = secondary vascular bundle; **TVB** = tertiary vascular bundle; **vb** = vascular bundle or collateral vascular bundle; **vbe** = vascular bundle with enlarged sheath. Not all are labelled. Scale bars: 0.3 mm.

Characters of the leaflet margin (Figure 3A):

1. Vascular bundles with enlarged sheaths (vbe) – These are a thick sclerenchymous fibre strands with a small portion of vascular tissue. They have been referred to as marginal veins somewhat enlarged (Tomlinson et al. 2011), vascular bundles with exaggerated sheaths (Noblick 2013, 2017) or vascular bundles with reinforced sclerenchymous sheaths (Sant’Anna-Santos et al. 2018). These are found on the edge of the leaflet margin. There are usually two on the leaflet margin with the larger one on the abaxial side. There are also one to three on the adaxial side of the mid-rib and often counted as part of the accessory bundles (ab).
2. Primary vascular bundles (PVB) – Primary vascular bundles are the largest vessels in the leaflet blade and connect to both the adaxial and abaxial hypodermis. They are a slightly swollen in the centre with large open xylem vessels with at least two to four poles of sieve elements and companion cells located abaxially.
3. Secondary vascular bundles (SVB) – Similar to primary vascular bundles but skinnier, also connected to both the adaxial and abaxial hypodermis, larger open xylem vessels are not clearly visible or nearly absent.
4. Tertiary vascular bundles (TVB) – Tertiary vascular bundles are paired vessels with one attached to the adaxial hypodermis and the other attached to the abaxial hypodermis. They are separated by two or more layers of mesophyll cells or chlorenchyma (cells with chlorophyll).
5. Miniveins (mv) – These veins are usually paired and located between the other three kinds of vascular bundles and are unattached to either the adaxial or abaxial surface, “floating” in the mesophyll. Occasionally, the adaxial one is missing. They often appear as two grey smudges under the microscope, one near the adaxial surface and one near the abaxial surface. The abaxial one is usually unattached or occasionally attached to the abaxial hypodermis and occasionally may have a few sclerenchymous cells. Miniveins are also the most common veins of the accessory bundles (ab) found in the mid-rib.

More characters for the leaflet mid-rib (Figure 3B):

1. Mid-rib fibrous ring (MFR) – This is a thick to thin sclerenchymous ring surrounding the veins or collateral vascular bundles (vb) in the centre of the mid-rib.
2. Collateral vascular bundles (vb) – These are vascular bundles composed of xylem and phloem that fill the centre of the mid-rib and are surrounded by the MFR.
3. Accessory Bundles (ab) – These are several mvs, miniveins and a few vbes, vascular bundles with enlarged sheaths, which partially or entirely surround the MFR in the mid-rib.
4. Phloem Poles (php) – These are clusters of sieve elements plus companion cells (phloem), either embedded within or adjacent to the abaxial and abaxial portion of the MFR in the mid-rib. They are always associated with xylem to form a vascular bundle (vb).

## Results

In the *Butia* leaflet margin (Figure 3A), the vessels are arranged in such a way that the adaxial (upper) and abaxial (lower) surface appear to “mirror” each other and is often referred to as isolateral symmetry. The primary, secondary and tertiary vascular bundles (PVB, SVB, TVB) appear to alternate with each other often in a repeating sequence. Miniveins (mv) are often found alternating with these larger vascular bundles. The leaflet margin itself is reinforced by at least two veins with enlarged fibrous sheaths (vbe). The larger vbe is usually located on the abaxial side of the leaflet. This isolateral anatomical leaflet symmetry is typical for the genus.

In the mid-rib (Figure 3B), there are usually one to three vbes present along the adaxial surface. Rarely are they absent. The centre mid-rib fibrous ring (MFR) is surrounded by several layers of parenchyma or chlorenchyma cells that form a proportionally thicker layer in juvenile leaves than in more mature and larger leaves. There are a number of accessory bundles (ab), mostly miniveins (mv) that are arranged within the chlorenchyma tissues and surround or partially surround the MFR. Most of the accessory bundles are unattached to the mid-rib surfaces, but include one to three vbes, which are attached. The MFR can be thick or thin depending on the age and maturity of the plant or from where the section was sampled. Small vascular bundles (vb) and phloem poles (php) can be found embedded within the MFR itself in older, mature plants, but are usually of a very small size and not easily detected in plants that are in a more juvenile state of development. Within the MFR are one or several vascular bundles (vb), sometimes called collateral bundles, consisting of xylem and phloem and they may or may not be orientated as adaxial and abaxial bundles. The largest vascular bundle is located abaxially in the mid-rib and is orientated with phloem or phloem poles embedded in the abaxial portion of the MFR and with well-developed xylem tissues just above. The other vascular bundles in the MFR are difficult to distinguish or even count, because they are not necessarily orientated as expected.

The proximal cross-sections are located between the centre and the base of the leaflet and the distal sections are located between the centre and the apex of the leaflet. By the same thinking, the proximal leaflets are located between the middle leaflet and the basal leaflet and the distal leaflets are located between the middle leaflet and the apex or leaf tip.

**Figure 4.**
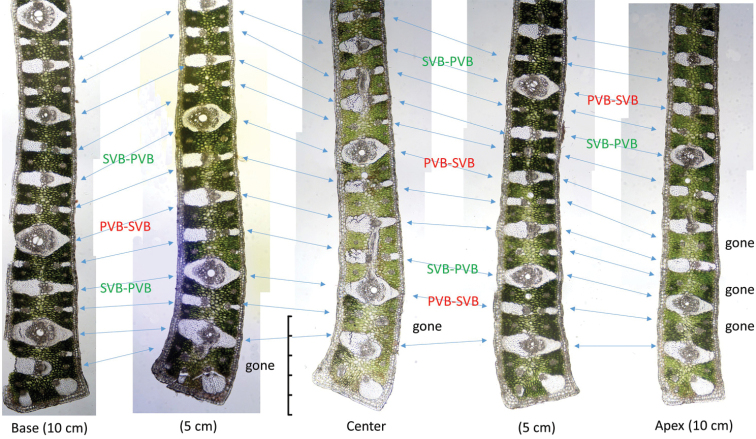
Anatomical changes of the leaflet margins in a 10 cm region to either side of the leaflet centre: *Butiaparaguayensis*. **PVB-SVB** = primary vascular bundles transitioning to secondary vascular bundles; **SVB-PVB** = secondary vascular bundles transitioning to primary vascular bundles. Gone = vascular bundle has disappeared or nearly ended. Scale bar: 0.5 mm.

**Figure 5.**
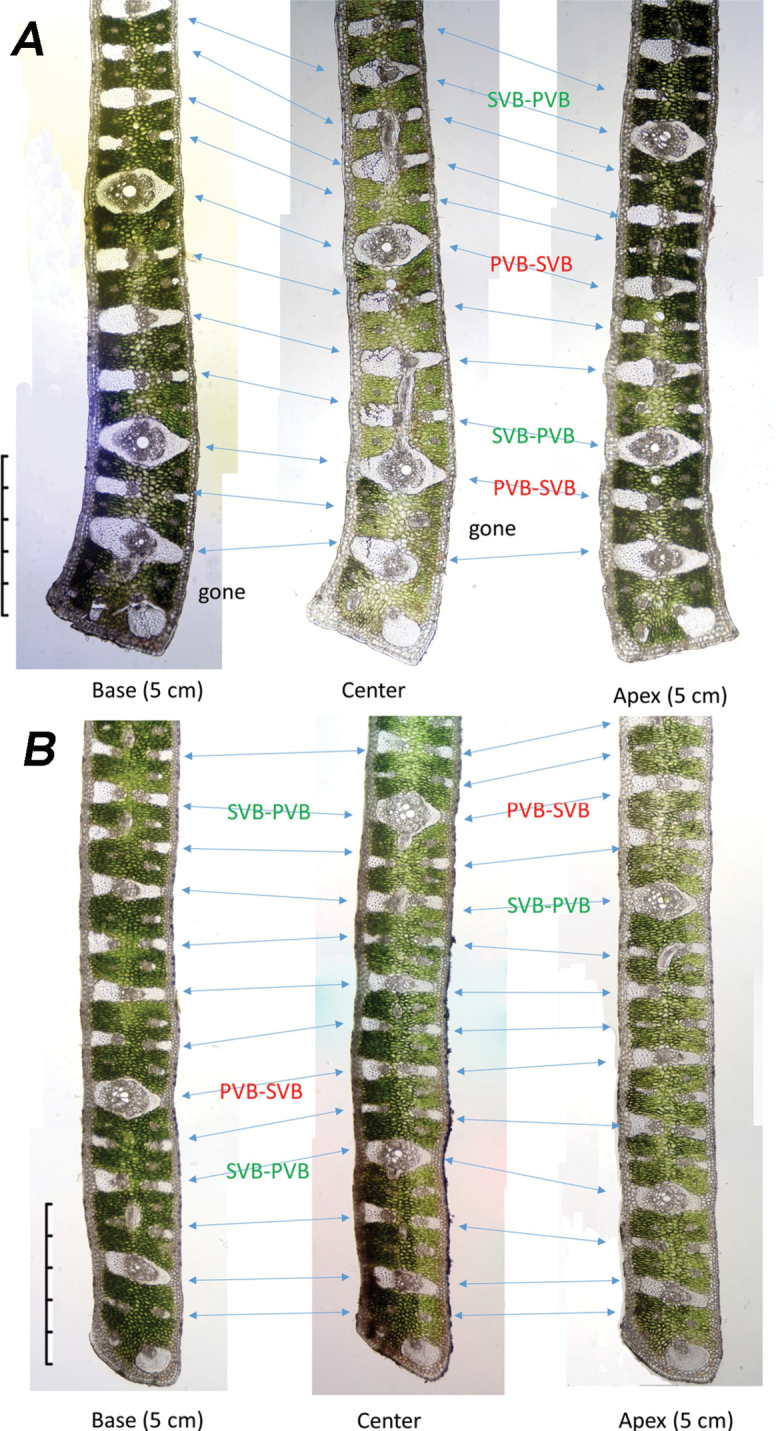
Anatomical changes of the leaflet margins in a 5 cm region near the leaflet centre **A***Butiaparaguayensis***B***B.eriospatha*. **PVB-SVB** = primary vascular bundles transitioning to secondary vascular bundles; **SVB-PVB** = secondary vascular bundles transitioning to primary vascular bundles. Gone = vascular bundle disappeared or nearly so. Scale bars: 0.5 mm.

**Figure 6.**
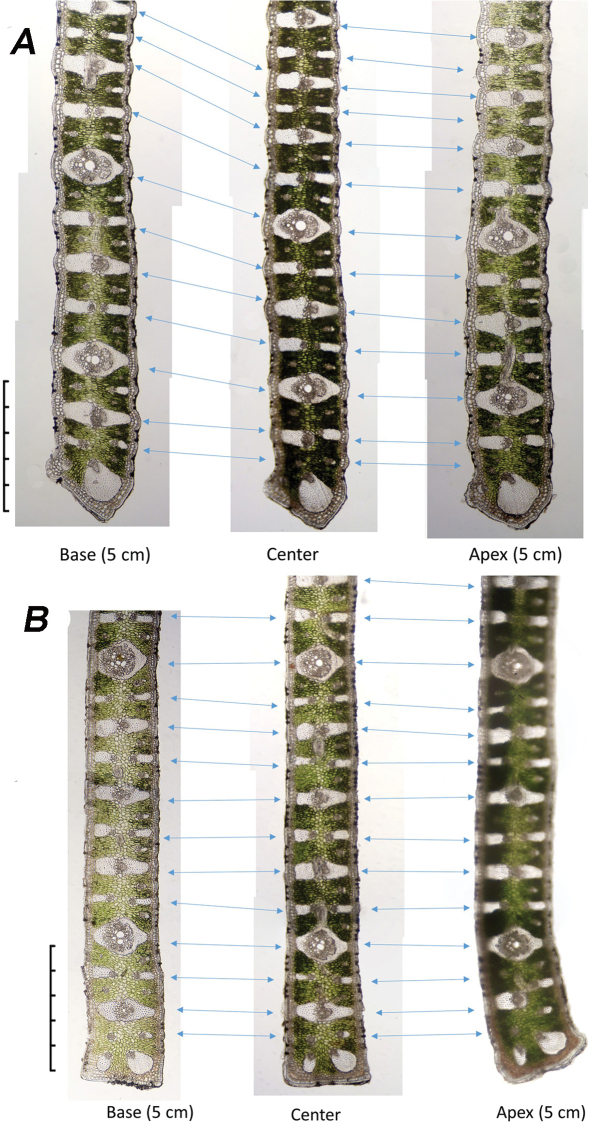
Anatomical changes of the leaflet margins in a 5 cm region to either side of the leaflet centre **A***B.yatay***B***B.odorata*. Note no transitional changes in these margins. Scale bars: 0.5 mm.

Table 1 summarises observations of variations that occur within the leaf margin of the four *Butia* species (Figures 4–6). We recorded changes between the centre cross-section and the cross-sections of the same leaflet made 5 cm to either side of it. Within that 10 cm length of leaflet margin, the changes observed include: a few of the vascular bundles do not extend between the base and apex of the leaflet as seen in *B.paraguayensis* (Figures 4, 5A). Most of the vascular bundles remain unchanged throughout as in *B.yatay* and *B.odorata* (Figure 6A, B), but in *B.paraguayensis* and *B.eriospatha*, a few change back and forth between primary (PVBs) and secondary vascular bundles (SVBs) (Figures 4, 5A, B). The tip of the margin remains unchanged in *B.eriospatha* and *B.yatay* (Figures 5B, 6A), but goes from adaxially prominent or bent to abaxially prominent towards the distal end (*B.paraguayensis*, Figures 4, 5A) or relatively unbent to abaxially prominent (*B.odorata*, Figure 6B).

**Table 1.** Changes that occur in the vascular bundles (veins) of the leaflet margins of four different *Butia* species from the proximal to the distal portion of the leaflet. Number of changes from primary vascular bundles (PVB) to secondary vascular bundles (SVB) and from SVB to PVB in that portion of the margin. Number of vascular bundles (VB) that disappear or nearly disappear (Figures 4, 5A, B, 6A, B).

Name (where sectioned)	PVB-SVB	SVB-PVB	VB nearly gone	Margin bent
*B.paraguayensis* (proximal 10 cm)				abaxially
*B.paraguayensis* (proximal 5 cm)	1	2	1	adaxially
*B.paraguayensis* (centre)			1	adaxially
*B.paraguayensis* (distal 5 cm)	2	2	1	abaxially
*B.paraguayensis* (distal 10 cm)	1	1	3	none
*B.yatay* (proximal 5 cm)				none
*B.yatay* (centre)				none
*B.yatay* (distal 5 cm)			1	none
*B.eriospatha* (proximal 5 cm)				none
*B.eriospatha* (centre)	1	2		none
*B.eriospatha* (distal 5 cm)				none
*B.odorata* (proximal 5 cm)				none
*B.odorata* (centre)				none
*B.odorata* (distal 5 cm)				abaxially

**Figure 7.**
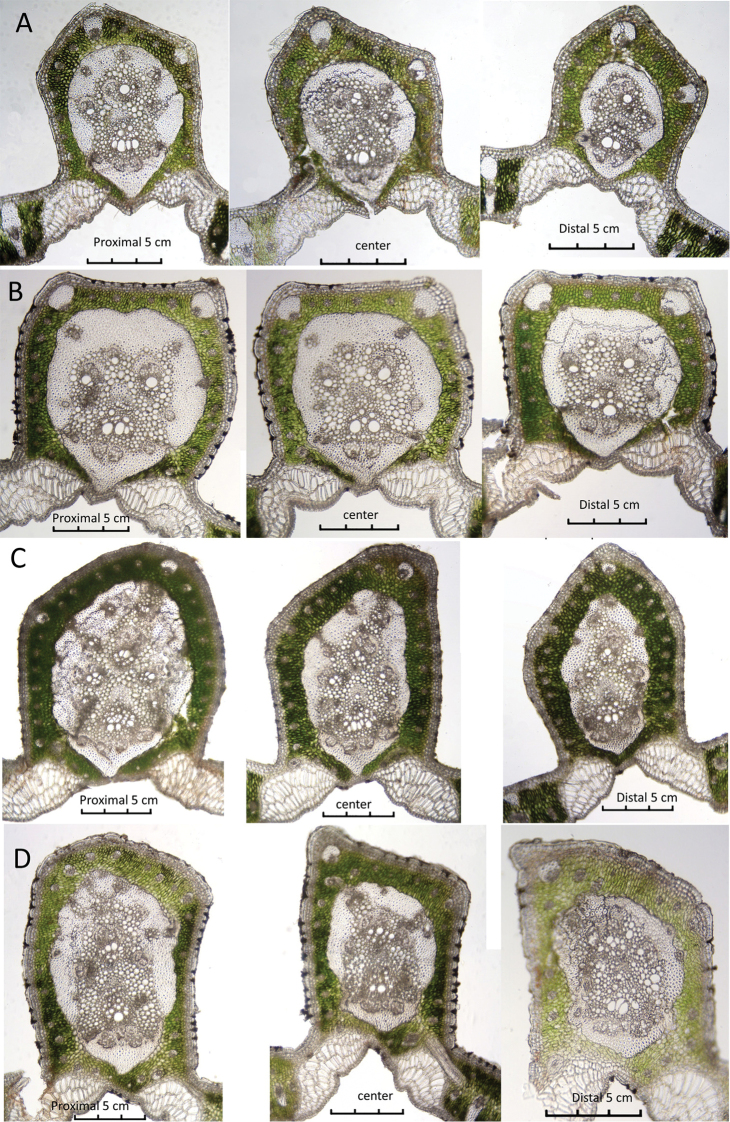
Within leaflet anatomical changes. Centre cross-section and cross-sections 5 cm to either side of it **A***Butiaparaguayensis***B***B.yatay***C***B.eriospatha***D***B.odorata*. Scale bars: 0.3 mm.

**Figure 8.**
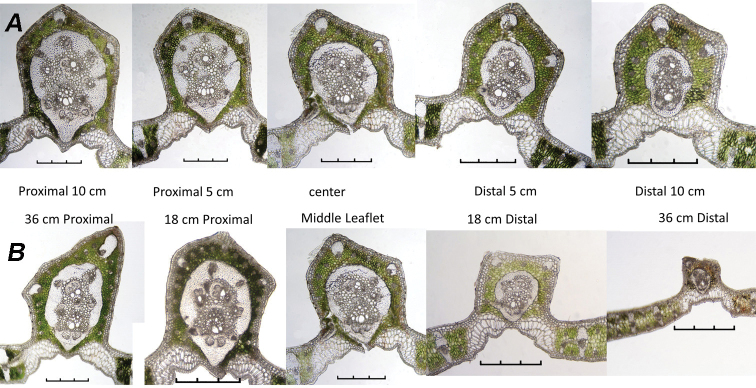
Within and between leaflet comparisons of *Butiaparaguayensis* cross-sections **A** centre cross-section and those sections 5 cm and 10 cm to either side of it within the same middle leaflet **B** centre of middle leaflet and centres of the leaflets 18 cm and 36 cm to either side of it. Scale bars: 0.3 mm.

Table 2 focuses on the differences found in the mid-rib within a single leaflet. The Table records the changes in the characters of the centre mid-rib section, the sections 5 cm to either side of it in all the species (Figure 7) and the sections 10 cm to either side of it in *B.paraguayensis* (Figure 8A). Mid-rib changes observed from proximal to distal ends of the leaflets are: the MFR becomes thinner; the MFR shape changes from abaxially projected to slightly projected (*B.yatay* and *B.eriospatha*, Figure 7B, C) to round (*B.paraguayensis* and *B.odorata*, Figure 7A, D); the MRF always reaches the hypodermis in *B.paraguayensis* and *B.yatay* (Figure 7A, B) and most of the time in *B.eriospatha* (Figure 7C), but almost never in the distal portion of *B.eriospatha* nor in any portion of *B.odorata* (Figure 7C, D). From the proximal to the distal end, there are fewer abs in the mid-rib and fewer vbs in the MFR in all species.

**Table 2.** Anatomical variation of the centre mid-rib cross-section of a middle leaflet, compared to proximal (prox.) and distal sections located 5–10 cm from the centre. ab = accessory vascular bundles, MFR = mid-rib fibrous ring, vb = vascular bundles or collateral vascular bundles, vbe = vascular bundle with enlarged sheath. (Figures 7, 8A).

Name (where sectioned)	MFR thickness (# cell layers)	MFR shape	MRF reaches hypodermis	#vbe	#ab	ab completely surround MFR	#vb in MFR
*B.paraguayensis* (prox. 10 cm)	9–11	ab-projected	yes	2	22	no	8
*B.paraguayensis* (prox. 5 cm)	8–9	ab-projected	yes	2	21	no	7
*B.paraguayensis* (centre)	6–7	slightly ab-projected	yes	2	17	no	5
*B.paraguayensis* (distal 5 cm)	4–5	round	yes	2	13	no	3–4
*B.paraguayensis* (distal 10 cm)	3–5	round	yes	2	9	no	3
*B.yatay* (proximal 5 cm)	6–10	ab-projected	yes	2	24	no	7
*B.yatay* (centre)	5–10	ab-projected	yes	2	21	no	6
*B.yatay* (distal 5 cm)	5–8	slightly ab-projected	yes	2	19	no	4
*B.eriospatha* (proximal 5 cm)	5–8	ab-projected	yes	2	26	no	9–10
*B.eriospatha* (centre)	4–7	ab-projected	yes	1	25	no	9
*B.eriospatha* (distal 5 cm)	2–7	slightly ab-projected	no	1	18	no	8
*B.odorata* (proximal 5 cm)	5–8	ab-projected	no	0	22	yes	8–9
*B.odorata* (centreer)	4–7	round	no	1	21	yes	5–6
*B.odorata* (distal 5 cm)	2–7	round	no	1	16	yes	5

**Figure 9.**
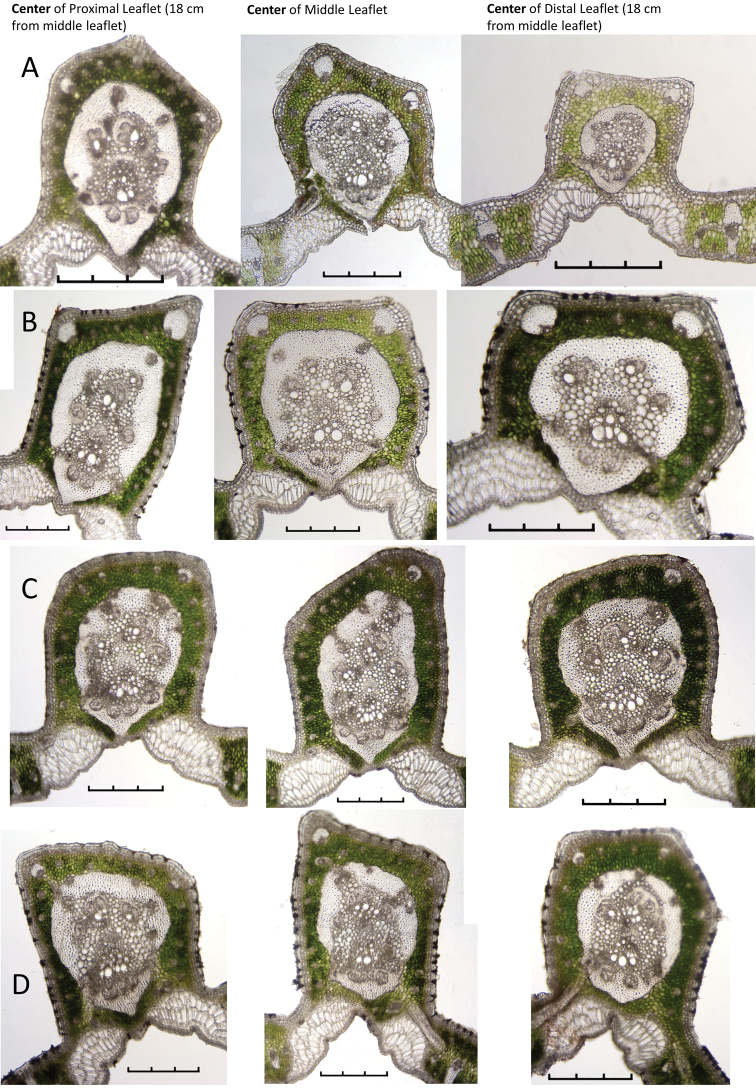
Between leaflet anatomical changes. Centre cross sections of middle leaflet of **A***Butiaparaguayensis* and the leaflets 18 cm to either side of it **B***B.yatay* and the leaflets 20 cm to either side of it **C***B.eriospatha* and the leaflets 20 cm to either side of it **D***B.odorata* and the leaflets 20 cm to either side of it. Scale bars: 0.3 mm.

Table 3 focuses on the differences found in the leaflet mid-rib cross-sections of the middle leaflet and those leaflets 18–20 cm to either side of it in all species (Figure 9) and the leaflets 36 cm to either side of it in *B.paraguayensis* (Figure 8B). We recorded the following trends from the proximal to the distal end of the leaf: in two of the species, the MFR becomes thinner (*B.paraguayensis* and *B.eriospatha*) (Figures 8B, 9A, C); three of the species have MFR shapes that change from abaxially projected to only slightly so (*B.yatay* and *B.odorata*, Figure 9B, D) or to almost round (*B.paraguayensis*, Figures 8B, 9A). From the proximal to the distal part of the leaf, all species have a reduced number of abs in the mid-rib. Finally, all usually have a reduced number of collateral vascular bundles, vbs, in their MFR.

**Table 3.** Anatomical variation of the centre mid-rib cross-section of a middle leaflet, compared to a proximal (prox.) and a distal leaflets located 18–20 cm from the middle and up to 36 cm from the middle in *B.paraguayensis*. ab = accessory vascular bundles, MFR = mid-rib fibrous ring, vb = vascular bundles or collateral vascular bundles, vbe = vascular bundle with enlarged sheath. (Figures 8B, 9).

Name (where sectioned)	MFR thickness (# cell layers)	MFR shape	MRF reaches hypodermis	#vbe	#ab	ab’s completely surround MFR	#vb in MFR
*B.paraguayensis* (prox. 36 cm)	8–9	ab-projected	yes	2	23	no	7
*B.paraguayensis* (prox. 18 cm)	8–9	ab-projected	yes	2–3	21	no	6
*B.paraguayensis* (middle)	6–7	ab-projected	yes	2	17	no	5
*B.paraguayensis* (distal 18 cm)	3–4	round	yes	2	10	no	3
*B.paraguayensis* (distal 36 cm)	2–4	round	yes	1	8	no	3
*B.yatay* (proximal 20 cm)	7–8	ab-projected	yes	2	25	no	7
*B.yatay* (middle)	5–9	ab-projected	yes	2	21	no	6
*B.yatay* (distal 20 cm)	6–8	slightly ab-projected	yes	2	16	no	5
*B.eriospatha* (prox. 20 cm)	3–7	ab-projected	yes	1	21	no	8–9
*B.eriospatha* (middle)	4–7	ab-projected	yes	1	25	no	9
*B.eriospatha* (distal 20 cm)	4–5	ab-projected	yes	2	20	no	6
*B.odorata* (proximal 20 cm)	6–7	ab-projected	no	2	22	yes	8–9
*B.odorata* (middle)	5–7	slightly ab-projected	no	2	21	yes	5–6
*B.odorata* (distal 20 cm)	4–5	slightly ab-projected	no	3	16	no	5

**Figure 10.**
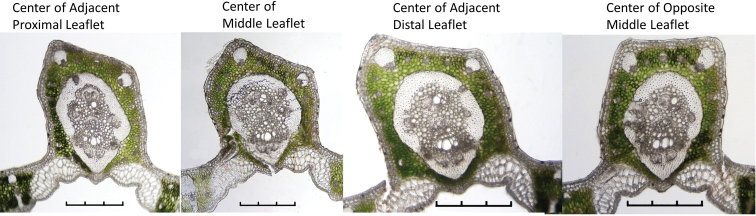
Between leaflet anatomical changes. Centre cross sections of the middle leaflet of *Butiaparaguayensis*, its two adjacent leaflets and the leaflet directly opposite it. Scale bars: 0.3 mm.

Table 4 summarises observations in *Butiaparaguayensis* comparing mid-ribs of the centre cross-sections of the middle leaflet, its two adjacent leaflets and the leaflet directly opposite it (Figure 10). We noted the following changes from the proximal to the distal portion of the leaf: the MFR changed from reaching the hypodermis to being separated from the hypodermis by at least one layer of chlorenchyma, from 21 accessory bundles (ab) to 15 and from six collateral vascular bundles (vb) to four (Figure 10). The middle leaflet on the opposite side has fewer abs (15 vs. 17) and fewer vbs (4 vs. 5) in the MFR than the selected middle leaflet (Figure 10).

**Table 4.** Anatomical variation of the centre mid-rib cross-section of a middle leaflet, compared to centres of the two adjacent (adj.) leaflets on the proximal and distal side and the centre of the opposite (opp.) middle leaflet. ab = accessory vascular bundles, MFR = mid-rib fibrous ring, vb = vascular bundles or collateral vascular bundles, vbe = vascular bundle with enlarged sheath. (Figure 10).

Name (where sectioned)	MFR thickness (# cell layers)	MFR shape	MRF reaches hypodermis	#vbe	#ab	ab’s completely surround MFR	#vb in MFR
*B.paraguayensis* (adj. proximal centre)	5–7	ab-projected	yes	2–3	21	no	6
*B.paraguayensis* (middle centre)	6–7	ab-projected	yes	2	17	no	5
*B.paraguayensis* (adj. distal centre)	5–7	round	no	2	15	no	3
*B.paraguayensis* (opp. middle centre)	4–6	ab-projected	yes	2	15	no	4

## Discussion

To our knowledge, there has never been a study that explored the anatomical changes that occur within a single palm leaf or even within a single palm leaflet. Most studies have been focused on the centre of the middle leaflet, which has been found to be very useful. This is certainly true for Glassman (1972), who pointed out that his *Butia* anatomy survey was based mostly on one specimen for each species and largely on material from the middle section of each leaflet. His study did not take into account any variation there may be amongst leaflets of different collections of the same species. Tomlinson (1961) examined and described the leaflet anatomy of 250 species of palms in 137 genera and suggested systematic relationships amongst genera. Tomlinson et al. (2011) expanded the original 1961 paper, presenting information on 183 palm genera of 185 now recognised and suggested relationships, based on anatomy and the use of modern phylogenetic approaches. Horn et al. (2009) took it a step further and mapped lamina anatomy on the phylogenetic tree for the palm family, based on plastid sequence data (Asmussen et al. 2006) in order to understand the evolution of lamina anatomy. Meerow et al. (2009) showed how leaflet anatomy supported the molecular relationships between *Allagoptera*, *Parajubaea* and *Polyandrococos* (now a synonym of *Allagoptera*). Glassman examined the anatomy of *Syagrus* and its closely-related genera (Glassman 1972, 1987). Noblick (2017) improved on Glassman (1972) by producing a key to identify 25 *Syagrus* species with short, subterranean stems (Noblick 2013) and a key to identify all 65 species and two subspecies of the genus, using only the anatomy of the leaflet margins (Noblick 2017). Noblick’s paper (2013) showed how leaflet anatomy could be used to support molecular data (Meerow et al. 2009).

Our study suggests that we need to take a step back and re-examine our previous data to reconfirm and re-evaluate the characters we have been using in our identification keys (Noblick 2013, 2017; Sant’Anna-Santos et al. 2018; Firmo et al. 2021) or in establishing our systematic relationships. As we have seen from this study, leaflet anatomy does vary even between leaflets that are literally adjacent to or directly opposite each other as in *Butiaparaguayensis* (Table 4, Figure 10). In the same leaflet, some species of *Butia* showed intraspecific variation in the leaf margin format (Sant’Anna-Santos et al. 2018), demonstrating the importance of studying more than one individual of the same species. By examining adjacent leaflets, Pinedo et al. (2016) discovered that the adaxial and abaxial prominent state of leaflet margins found in *Allagoptera* are simply complementary conditions found between two adjacent leaflet margins. Their work showed the importance of examining adjacent leaflets.

The leaf margins of *Butia* species contain few characters to distinguish species, but the vasculature within each leaflet changes more frequently in some species than in others over a 10 cm length. Changes were observed to take place in *B.paraguayensis* and *B.eriospatha* (Figures 4, 5A, B) with secondary vascular bundles (SVB) becoming primary vascular bundles (PVB) and vice versa, but with no or few changes seen in *B.yatay* and *B.odorata* (Figure 6A, B). Some vessels appear to have disappeared from one section to the other in *B.paraguayensis* (Figures 4, 5A). However, future studies in which refined techniques are employed in sample preparations will allow thin paradermal sections that are essential in understanding the vascular pattern in *Butia*.

**Figure 11.**
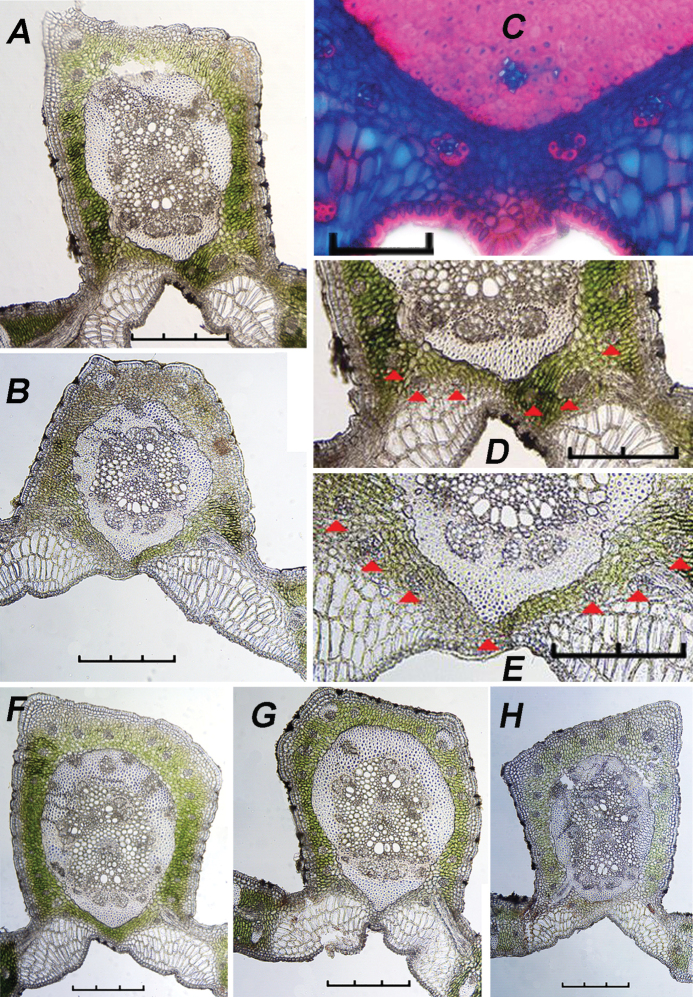
Population and individual differences in *Butiaodorata***A, B, D–H** are from Rocha, Uruguay, **C** is from Tapes, RS, Brazil **A** 20060233*E **B** 20060233*G **C** contrasting stains make the abs from the Brazil specimen more visible **D** enlargement of A **E** enlargement of B **F** 20060234*M **G** 20060237*C **H** 20060240*A. Note that the presence of accessory bundles (ab) surrounding the mid-rib fibrous ring (MFR) is more apparent in the Brazil sample C partially due to the staining, but less apparent or absent from the Uruguay samples. Red arrows pointing out the abs in the Uruguayan samples and abs do not completely surround the MFR in **F–H**. Scale bars: 0.3 mm (**A, B, F–H**), 0.1 mm (**C**), 0.2 mm (**D, E**).

Amongst several characters used by Sant’Anna Santos et al. (2018), they used the following in their key: the number of mid-rib collateral bundles (vb), the mid-rib connected or not connected to the hypodermis, the mid-rib fibrous ring (MFR) abaxially projected or not projected and accessory bundles (ab) completely surrounding the MFR or only partially surrounding it to identify *Butia* species. As seen in the Tables, many of these characters do vary, with the number of collateral bundles decreasing in number from the base to the apex, whether that is within the leaflet or between leaflets (see #vb in MFR in Tables 2–4). Whether the MFR is connected to or reaches the hypodermis or not is variable in at least the adjacent distal leaflet of *Butiaparaguayensis* (Table 4, Figure 10) or in the distal portion of the leaflet of *Butiaeriospatha* (Table 2, Figure 7C). The shape of the MFR changes from abaxially projected and slightly projected to round from the base to the apex, whether that is within a leaflet or between leaflets (see MFR shape in Tables 2–4, Figures 7–10). Accessory bundles (ab) completely surrounding the MFR is one of the main characters used to distinguish *Butiaodorata* from *Butiacapitata* (Sant’Anna-Santos et al. 2015). This was clearly the case for the samples of five specimens from Tapes, RS, Brazil. Of course, accessory bundles cannot completely surround the MFR if they are interrupted by the MFR being connected to the abaxial hypodermis, as in *B.capitata* from Lontra, MG (Sant’Anna-Santos et al. 2015). In our study, the MFR is not connected to the abaxial hypodermis in most specimens of *Butiaodorata* from Uruguay, just like the specimens from Tapes, RS, studied by Sant’Anna-Santos et al. (2015). While on close examination the accessory bundles (ab) of some Uruguayan specimens (Figures 7D, 11A, B, D, E) do encircle the MFR (fibrous ring) completely as do those of the more northern populations from Tapes, RS (Figure 11C) (Sant’Anna-Santos et al. 2015), it is not strongly apparent as seen in specimens from Tapes, RS. In fact, in many Uruguayan specimens of *B.odorata*, this is clearly not the case, because while the abs appear to mostly encircle the MFR, they do not completely surround it (Figure 11F–H). So the key will have to be modified to accommodate these population differences. In addition, note that Figure 11A, B are the same 20060233 accession, meaning that both plants were grown from seed collected from the same mother plant. Therefore, it is important to be cognizant of not only population differences when researching anatomical variation, but even intra-plant differences, as previously stated by Sant’Anna-Santos et al (2018) in the leaflet margin of some species of *Butia*. In most cases, the anatomy is consistent. Sant’Anna-Santos et al. (2018) studied both cultivated and native *Butiacapitata* and *Butiaarcheri* specimens and found the same anatomical organisation regarding the leaf anatomy. Although variation does not seem to be a rule for all species, our data show that it is important to investigate different populations of the same species.

**Figure 12.**
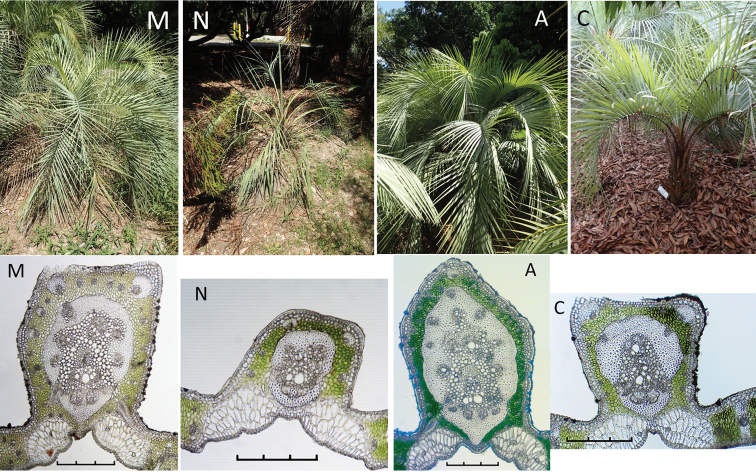
Differences in the centre mid-rib cross-sections within plants of the same species and same accession (seed collected from the same mother plant), but growing under different environmental conditions resulting in a different stage of developmental plant and leaf maturity **M, N** are *Butiaparaguayensis* plant accession 20060222*M and 20060222*N **A, C** are *Butiayatay*. plant accession 20040335*A and 20040335*C. Note the differences in the developmental stage of the plant above and its corresponding anatomy below.

Montgomery Botanical Center has several wild collected plants that were grown from seed collected from the same mother. The plants, however, have developed differently due to their unique set of growing conditions, like sun/shade exposure, differences in soil type and available moisture. Certainly, the use of fertilisers may be responsible for favouring the growth of vegetative organs and thus modifying the basic histological patterns. It has been reported that *Butiapubispatha*, under cultivation, showed an accelerated growth, resulting in larger plants than those observed in its natural habitat (Lorenzi et al. 2010). The different growing conditions at MBC resulted in some palms growing more robustly and maturing faster than others. Although it was not an objective of this current study, it is important to note that different growing conditions also result in plants, even from the same mother, having different mid-rib anatomy. Figure 12 shows the images of palms with their corresponding anatomy below. The *B.paraguayensis* labelled specimen M is obviously a larger and more mature specimen than N of the same accession and also the *B.yatay* specimen A is larger and more mature than C. The resulting differences in their anatomy M to N and A to C illustrate the importance of knowing the condition of the palm from which you are sampling, because it does make a difference. Finally, although the anatomical results of plants grown from wild collected seed should not be affected greatly from those collected directly from the wild, the progeny of these may experience changes due to the potential for hybridisation in cultivation.

## Conclusion

This paper has shown the importance of always collecting leaflets as close to the middle of the leaf blade as possible and sectioning that leaflet as close to its centre as possible for consistent and comparable results. It is also important to expect some population differences, differences in plants of different developmental maturity and differences in those growing under distinctly different conditions than those found in their original habitat. Here, we re-emphasise the importance of a broader sampling exercise when studying leaf anatomy due to possible ecological and developmental variations that may occur in some species. The diversity of leaf anatomy, here observed, also led us to suggest that characters previously used should be re-evaluated in further studies, using wild populations and/or cultivated specimens.
